# Supplementary material for: Untargeted metabolomics of COVID-19 patient serum reveals potential prognostic markers of both severity and outcome
Source: Metabolomics. 2021 Dec 20;18(1):6. doi: 10.1007/s11306-021-01859-3 (PMC8686810; doi:10.1007/s11306-021-01859-3)
Supplement: Supplementary file 1 — Supplementary file1 (DOCX 1831 kb) [file 11306_2021_1859_MOESM1_ESM.docx]

Supplementary information: Untargeted metabolomics of COVID-19 patient serum reveals potential prognostic markers of both severity and outcome.

# Supplementary results and figures

**Table S1:** **Overview of findings and study design in published studies utilising metabolomics to assess COVID-19 diagnosis, severity, and outcome up to May 2021.** Abbreviations: MSML IROA, Mass Spectrometry Metabolite Library supplied by IROA Technologies; GC-MS: Gas Chromatography-Mass Spectrometry; AEX-LC-MS/MS: Anion Exchange-Liquid Chromatography-Tandem Mass Spectrometry; NMR: Nuclear Magnetic Resonance.

| **Reference** | **Patient groups and cohort sizes** | **Outcome** | **Metabolites/Metabolic pathways** | **Targeted/untargeted** |
| --- | --- | --- | --- | --- |
| (Blasco et al., 2020) | Plasma from time of COVID-19 diagnosis based on SARS-CoV-2 PCR for COVID-19 patients vs control patients (does not provide details).  Patient numbers: 55 COVID-19 patients vs 45 controls | Predict: diagnosis from metabotype and COVID-19 disease evolution (Day 7 and Day 15) | Cytosine and tryptophan metabolism as marker of SARS-CoV-2 infection and COVID-19 severity/outcome | Targeted LC-MS based on MSML, IROA |
| (Shen et al., 2020) | Serum from patients with COVID-19 diagnosis vs patients with similar symptoms but negative SARS-CoV-2 RTPCR or healthy control subjects.  Patient numbers:   - 28 severe COVID-19 - 25 non-severe COVID-19 - 28 healthy subjects - 25 non-COVID-19 (COVID-19 symptoms, negative test) | Differences in proteomic and metabolomic profiles of COVID-19 vs healthy controls. | Dysregulated lipid, amino acid and kynurenine metabolism. Increased kynurenine pathway metabolite levels in COVID-19 patients  Reduction in levels of many lipids in COVID-19 patients vs controls  Reduced levels of amino acids and derivatives, particularly metabolites of arginine metabolism, in COVID-19 patients vs controls | Untargeted LC-MS/MS |
| (Overmyer et al., 2020) | Plasma on hospital admission with moderate to severe respiratory issues with suspected SARS-CoV-2 infection. Those with negative COVID-19 test outcome assigned to controls.  Patient numbers: 102 COVID-19 vs 26 non-COVID-19 (COVID-19 symptoms, negative test) | Prediction of COVID-19 severity along with proteomic and transcriptomic data | Lipids and tryptophan metabolites associated with COVID-19 severity. | GC-MS and AEX-LC-MS/MS. Semi-targeted |
| (Thomas et al., 2020) | Serum samples. Day of sample draw not specified. Some controls were COVID-19 convalescent patients (negative for SARS-CoV-2 RTPCR)  Patient numbers: 33 COVID-19 vs 16 controls | Observational study | Alterations in metabolism of lipids, amino acids (particularly tryptophan and arginine) in COVID-19 patients. | Untargeted and targeted LC-MS/MS |
| (Kimhofer et al., 2020) | Plasma from 17 adults with positive SARS-CoV-2 test result vs 25 healthy controls from general population | Differentiate SARS-CoV-2 infected patient from controls | Alterations in lipid and amino acid metabolism, particularly tryptophan metabolism in COVID-19 vs healthy patients. | NMR and targeted LC-MS of amino acids |
| (Wu et al., 2020) | Plasma of 10 healthy controls and 34 COVID—19 patients (9 fatal, 11 severe and 14 mild). Longitudinal samples were included in the study. | COVID control comparison, severity, and outcome | Metabolic and lipid alterations linked to the course of the disease. More particularly change sin malic acid levels, carbamoyl phosphate and guanosine monophosphate. | Untargeted LC-MS |
| (Páez-Franco et al., 2021) | Serum from 46 severe and 19 mild COVID-19 patients | Compare controls, mild and severe COVID-19 patients. | Alterations of amino acid metabolism and Warburg effect in COVID-19 patients that increases with the severity of the disease. | GC-MS |
| (Sindelar et al., 2021) | Plasma samples from 341 individuals from which 274 COVID-19 patients. Longitudinal samples were gathered for a subset of the initial patients group. | Severity predictive model and longitudinal evolution. | 25 predictive compounds (22 lipids and 3 polar metabolites). | Untargeted LC-MS for polar metabolites and lipids dedicated methods. |
| (López-Hernández et al., 2021) | Plasma samples of 161 symptomatic individuals from which 39 controls, 40 COVID-19 non-hospitalized patients, 42 hospitalized COVID-19 patients that did not require intubation and 40 COVID-19 patients that require intubation. | Predictive models distinguishing between:   - Control vs mild COVID-19 cases - Hospitalized vs non-hospitalized COVID-19 patients - Intubated vs non-intubated severe COVID-19 cases | Kynurenine:tryptophan ratio, long-chain saturated and monounsaturated LysoPC so as butyric and pyruvic acids | Targeted LC-MS |
| (Ansone et al., 2021) | Serum of 32 hospitalized COVID-19 patients. Analysed sample at admission and recovery phase. | Metabolite levels in acute phase compared to recovery | L-glutamine reduced in acute phase. Citrulline and ornithine reduced in acute phase. | Targeted LC-MS |
| (Doğan et al., 2021) | Serum from 44 COVID-19 patients and 41 controls | COVID-19 vs controls | Unregulated purine,  Downregulated glutamine  Changes in leukotriene D4 (LTD4), and glutathione metabolisms | Untargeted LC-MS |


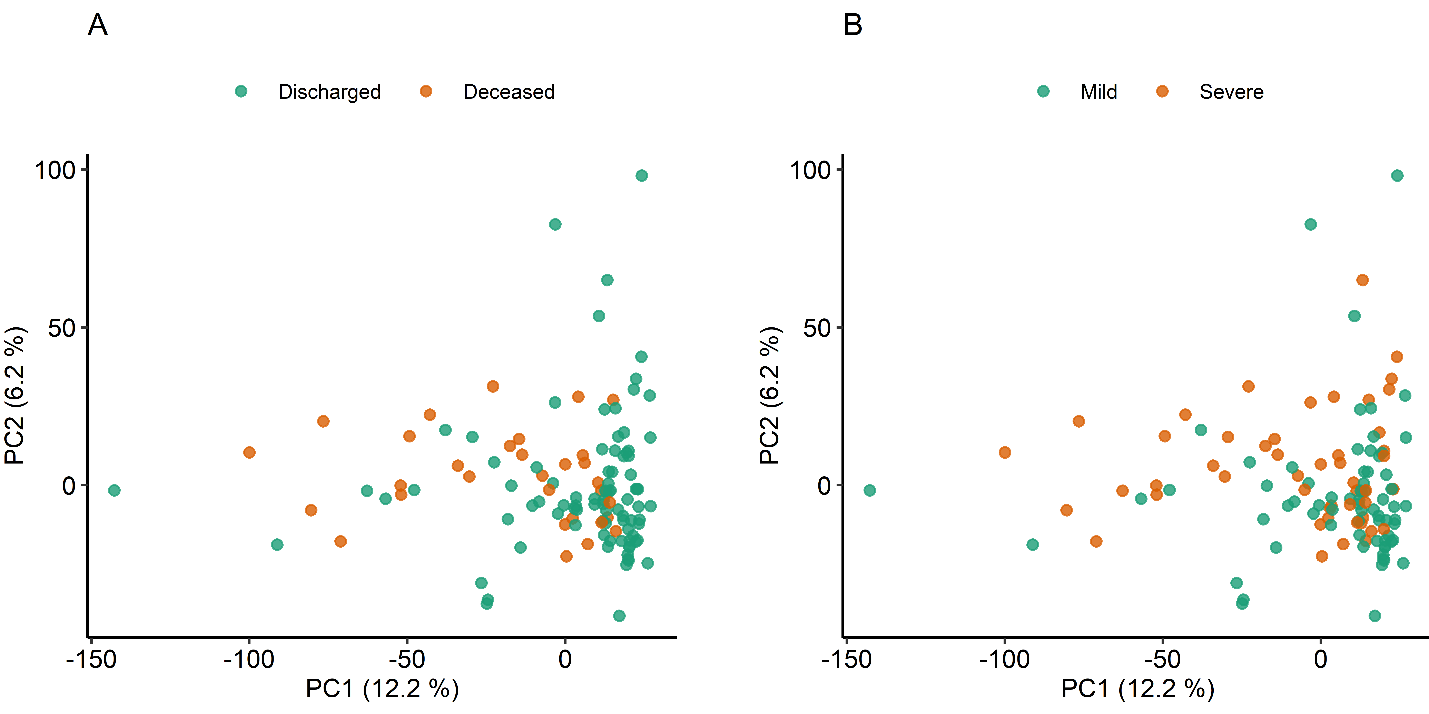


**Figure S1: PCA scores plot of the 120 patients coloured by outcome(A) and severity(B) in ESI+.** No clear group separation is visible. However, mild cases and discharged patients tend to cluster in the bottom right corner. Moreover, we can note that only a relatively small percentage of the variance is explained by the first two components. Axes are labelled with principal component and its explained variance in percentage.


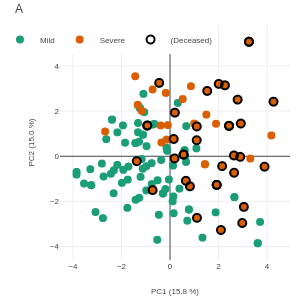

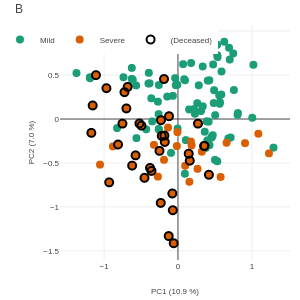


**Figure S2:** **Metadata alone (A) and multiblock (B) PCA for severity and outcome.** Group separation trends improve with addition of LC-MS data (B). It is important to note that deceased patients are a subset of severe cases that do not appear to show specific clustering in this exploratory analysis. Axes are labelled with principal component and its explained variance in percentage.


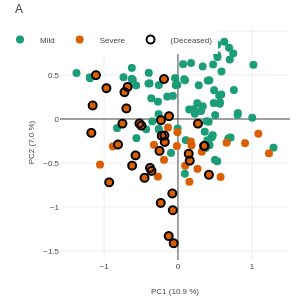

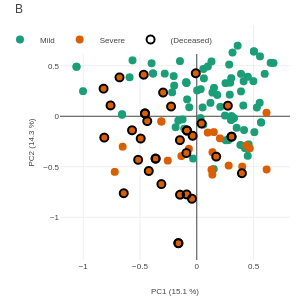


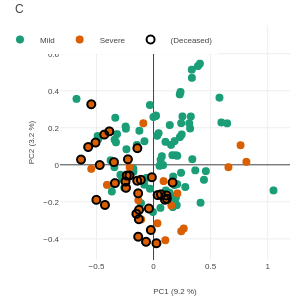

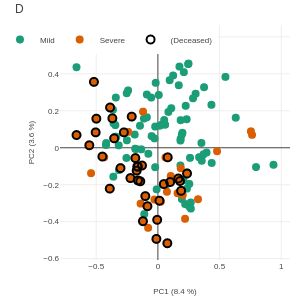


**Figure S3: Multiblock PCA in severity and outcome.** Super scores plot (A), followed by individuals blocks scores with metadata (B) LC-MS ESI+ (C) and LC-MS ESI- (D). Axes are labelled with principal component and its explained variance in percentage.


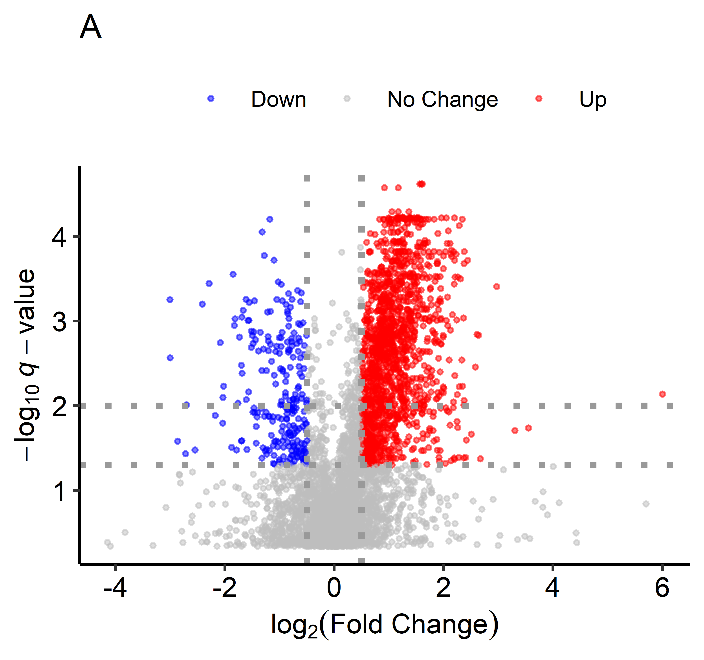

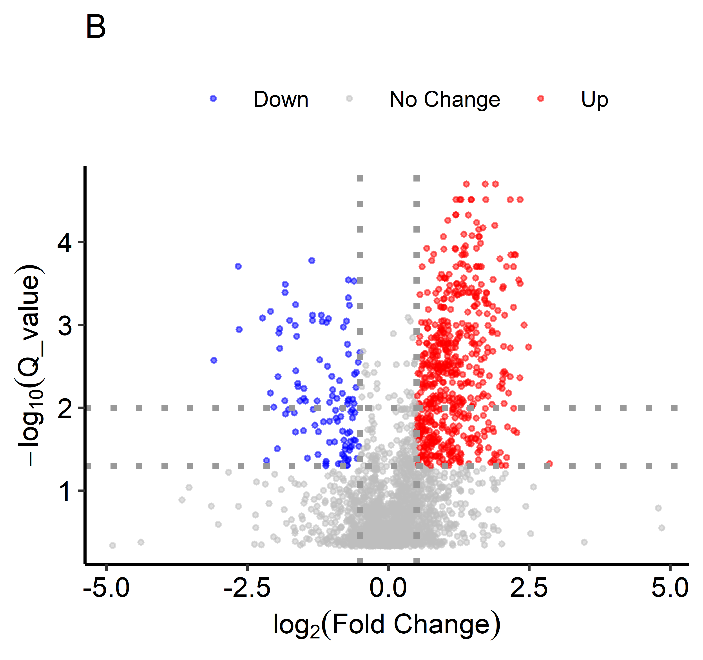


**Figure S4:** **Volcano** **plots showing metabolomic features discriminating disease outcome in ESI+(A) and ESI-(B)**. Dotted lines mark boundary of significance (0.5 for Log_2_ Fold Change (FC) and 2 lines for q-value at 0.05 and 0.01). Metabolic features with higher levels in poor outcome are coloured in red and inversely metabolic features with significantly lower levels in blue. In both ESI+ and ESI-, differences in fatal outcome tended to be more often based on increased levels.


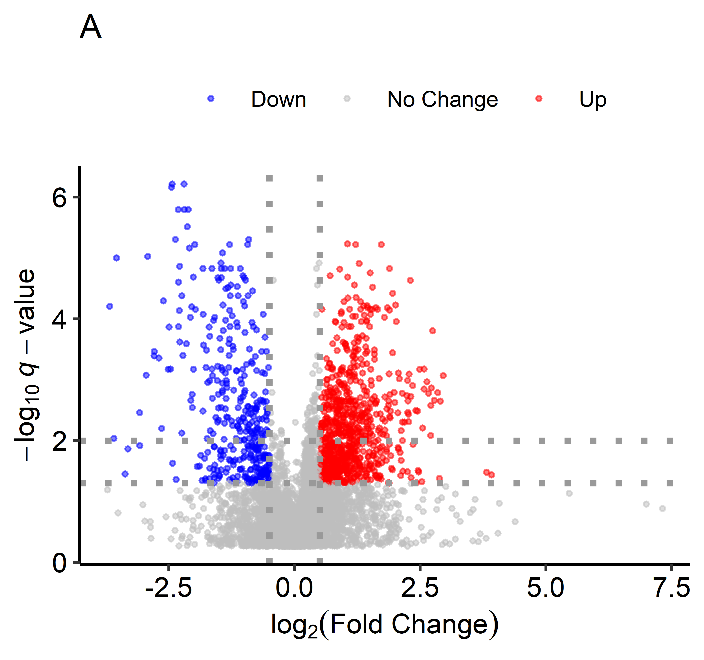

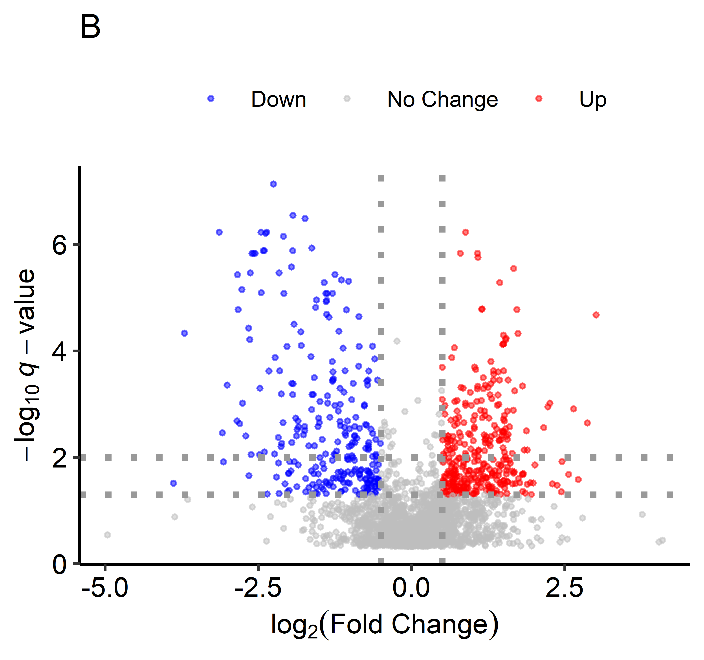


**Figure S5: Volcano plots for metabolomic features discriminating infection severity in ESI+ (A) and ESI- (B).** Dotted lines mark boundary of significance (Log_2_ FC at 0.5 and q-value at 0.05 with second dotted line at q-value at 0.01). Metabolic features with higher levels in poor outcome are coloured in red and inversely metabolic features with significantly lower levels in blue.

**Table S2:** **Number of metabolic features remaining after LC-MS/MS data pre-processing, statistical selection of significant features and manual curation for chromatographic peak shape and spectral signal.** Metabolic features where no molecular formula could be assigned by CD3.1 (see Methods) were discarded. Mass library match refers to match of the calculated molecular formula and monoisotopic mass within 3ppm.

| **MSI level (Sumner et al., 2007)** | **ESI+** | **ESI-** |
| --- | --- | --- |
| **Level 4: Molecular formula match** | 526 | 409 |
| **Level 3: Molecular formula + Mass library match** | 384 | 283 |
| **Level 2: all above plus MS/MS match (spectral match ≥ 70%)** | 45 | 33 |
| **Level 1: MS, MS/MS and RT match to experimental in-house library** | 14 | 8 |


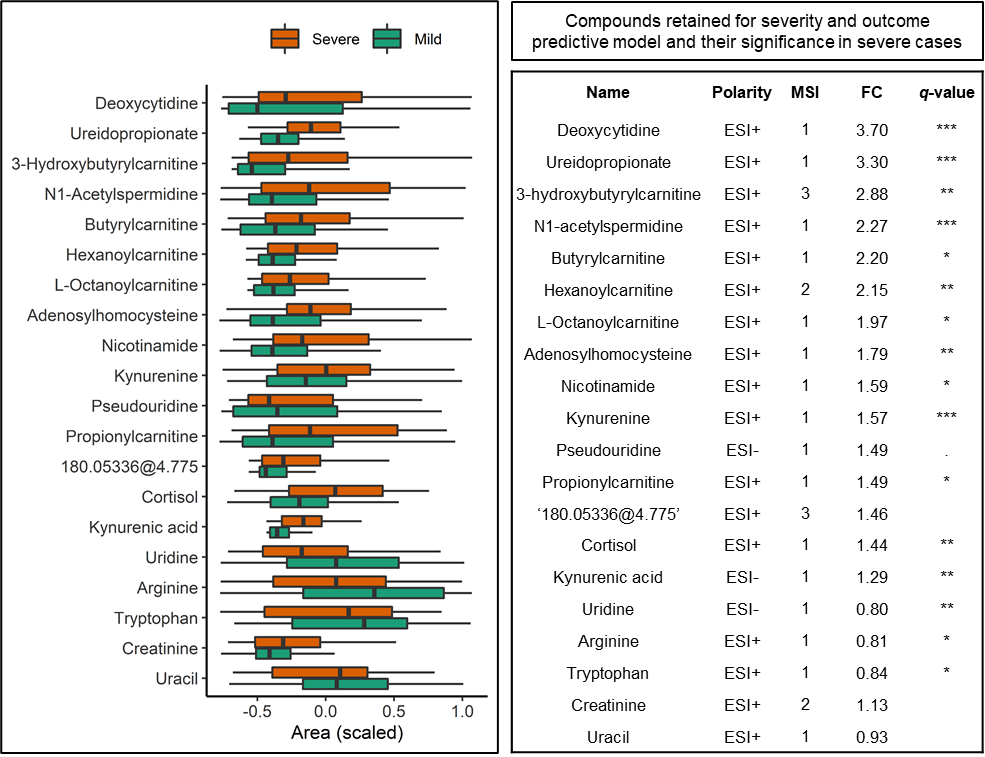


**Figure S6: Compounds retained for the predictive model and their significance in severity.** Box plot shows compound area differences between mild and severe COVID-19 patients ordered by fold change. Compound areas are standardized (mean = 0, SD = 1) to facilitate comparison. Boxes represent the quartiles Q1 to Q3 with Q2 (i.e., median) line in the middle. The ‘whiskers’ depict the upper and lower limit i.e., Q1 ± (Q3-Q1). For visualization simplicity the data is clipped between 10 and 90 percentiles. The table on the right side of the figure shows detailed information about the compounds including FC and q-value (false discovery rate corrected p-value) following ‘star’ notation i.e., ‘***’ correspond to q-values <0.001, ‘**’ <0.01, and missing when >0.1.


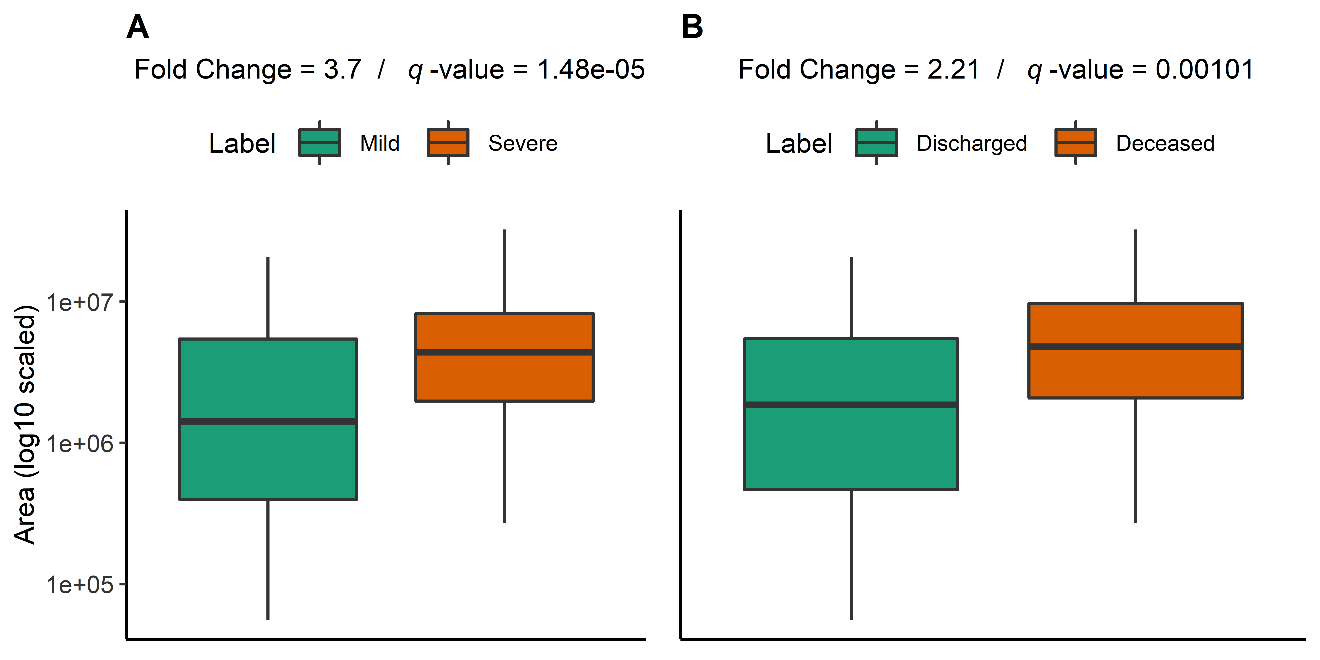


**Figure S7:** **Deoxycytidine levels in patients according to (A) severity or (B) outcome.** Boxplot markings follow the same standard as Figure S6.

**Table S3: Adjusted logistic regression results by severity.** Positive OR indicate increased levels in patients with poor outcome. OR are presentred with OR(95%CI). Significance is presentedin ‘star’ notation i.e. ‘***’ correspond to p-values <0.001, ‘**’ <0.01, ‘*’ <0.05, ‘.’ < 0.1 and missing when >0.1.

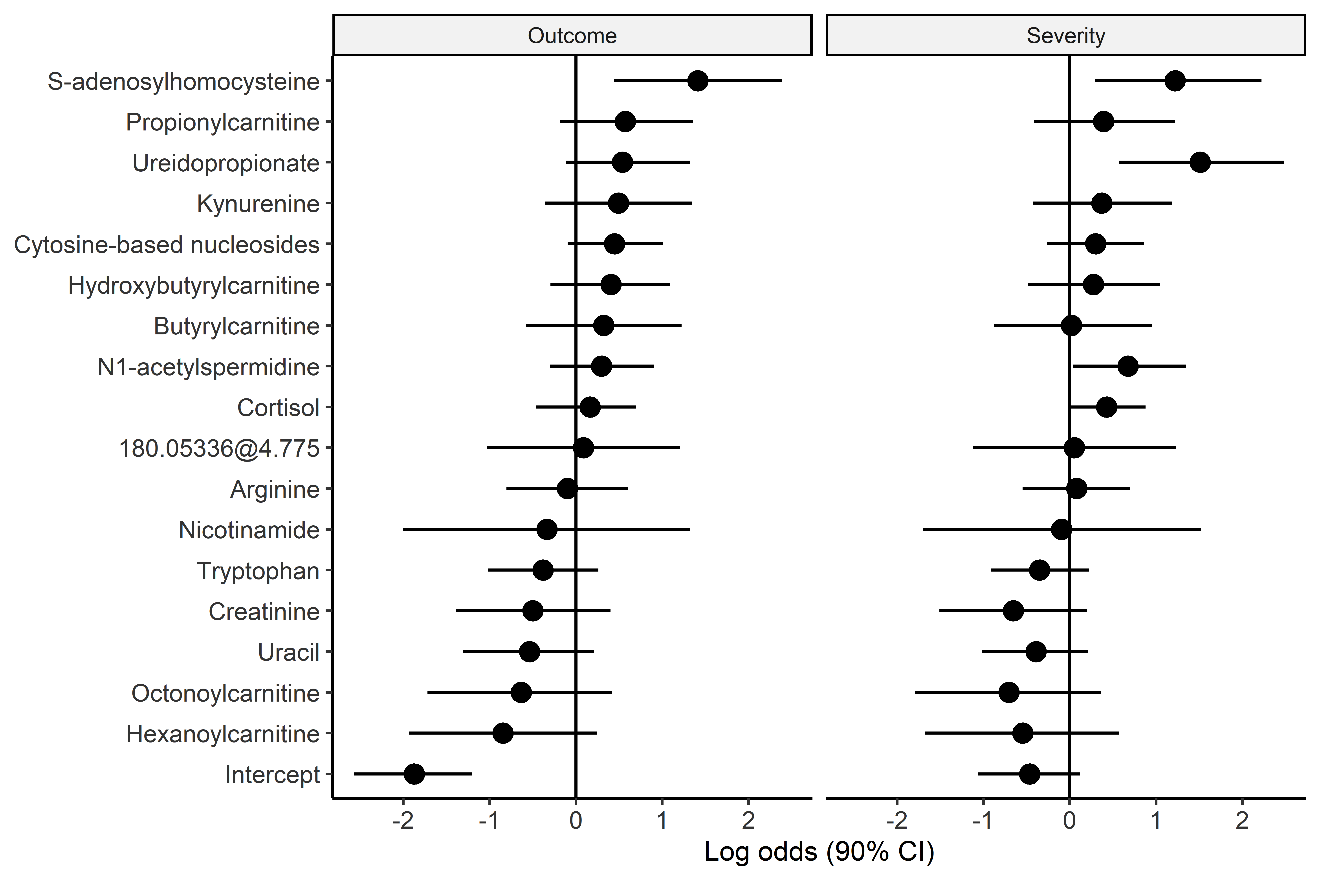


**Figure S8:** **Plot of log odds with 90% posterior CI for both outcome and severity model ordered by mean log odds in outcome.** These parameters are from the multivariate model and the log odds CI are not meant to indicate individual significance, but to illustrate alignment in directionality between outcome and severity models. Intercept in the figure refers to the intercept of the generalized linear model, in this case the log odds when all predictors are equal to their mean as data is standardized (mean = 0, SD = 1).

## Compound identity validation

Compound identity validation was performed when necessary to establish MSI 1 level for all selected compounds. Validation criteria were based on fragmentation spectra similarity (i.e., fragments and intensity levels) and overlap in RT in the LC method used. Where challenges were encountered e.g., deoxycytidine and ureidopropionate, details are presented in in this section and pooled serum from the study was spiked with standards to establish clear RT overlap in matrix.

### Deoxycytidine

Cytidine and deoxycytidine fragmented at the ESI source resulting in cytosine events on the LC separated by approx. 0.1 min for cytosine, cytidine and deoxycytidine respectively as demonstrated in Figure S9. This indicated that the N-glycosidic bond between the cytosine and ribose moiety breaks easily. Therefore, we analysed the cytosine source fragmentation in all cytosine-based nucleoside, ribonucleotides and deoxyribonucleotides. All of these except CDP and CTP showed loss of cytosine however, only cytidine and deoxycytidine had strong signal in ESI+. Figure S10 shows pooled study serum spiked with 5 µM of the chemical standard that overlays perfectly with the 3 cytosine events detected in the study samples. Study serum cytosine levels were particularly low and undetected in some patients leading to a possible confusion of cytidine or deoxycytidine detection.

In the case of deoxycytidine approximately 2/3 of the molecules fragment at the source (see Figure S11) making the cytosine event a more reliable measurement for the deoxycytidine levels. Therefore, in the discovery study we used cytosine event areas at the deoxycytidine retention time for all reported results. In the validation study all three cytosine events i.e., cytosine, cytidine and deoxycytidine were summed up together and are referred as cytosine-based nucleosides. At this stage of the study this level of precision is sufficient and allows simpler and more accurately comparable measurements.

### Ureidopropionate

Ureidopropionate tends to fragment at the ESI source leading to low levels of precursor ions and unreliable MS/MS acquisition. Its elution time follows that of isobaric asparagine by 0.2 min leading to possible confusion of the two compounds. A parallel reaction monitoring (PRM) method was used to capture the fragmentation profile to confirm the identity of ureidopropionate by MS/MS fragmentation spectra in addition to chemical standard-based RT validation. The PRM obtained MS/MS spectra showed signs of background ions that were filtered out based on mass defect and a coeluting ion in the serum. However, the two identifying fragments for ureidopropionate, 90.0550 and 115.0502, showed a linear response with increasing sample injection volumes (1, 5 and 10 µL).


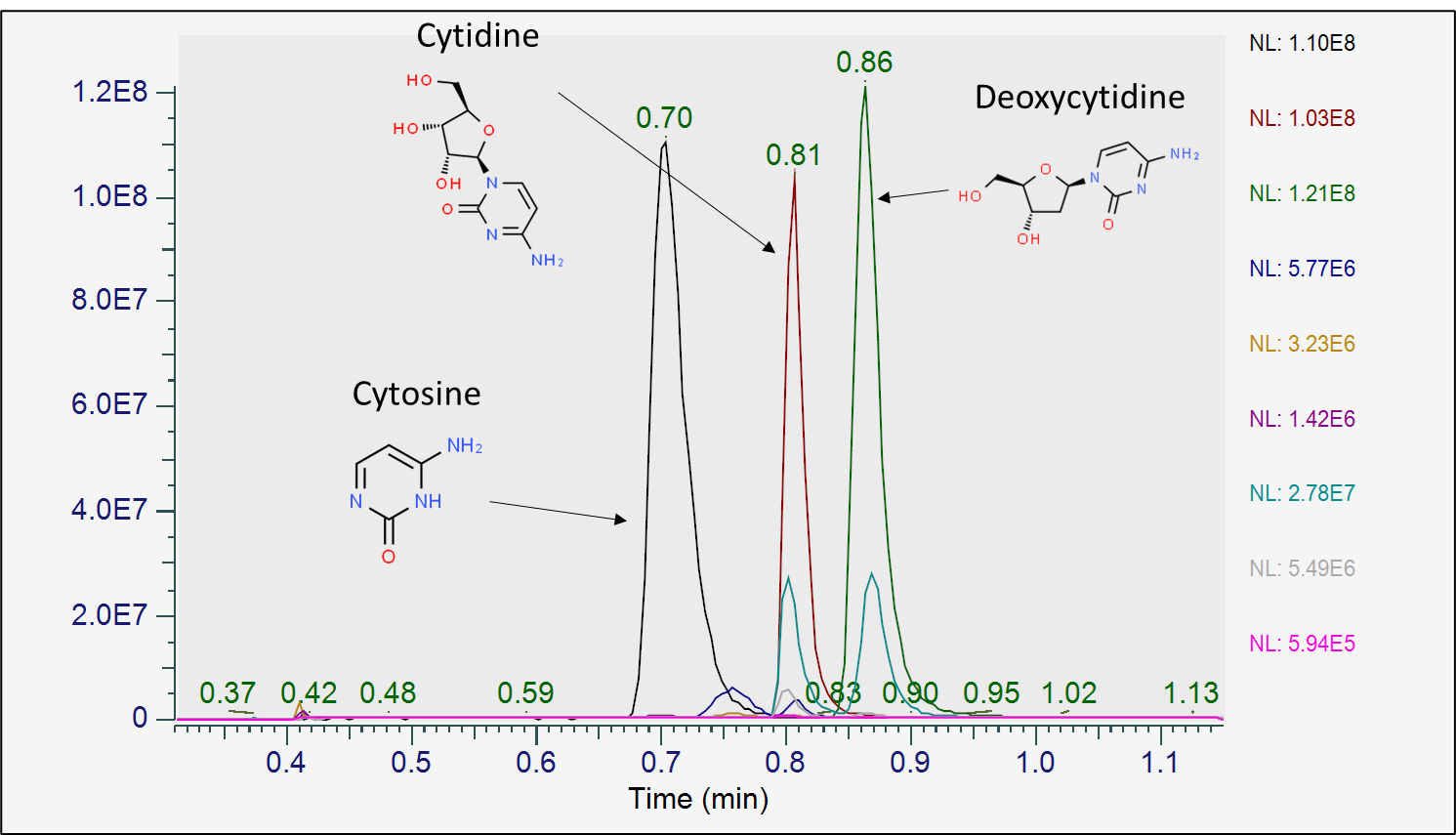


**Figure S9: Cytosine LC-MS ESI+ event from cytosine-based nucleoside, ribonucleotides and deoxyribonucleotides**. LC-MS and LC-MS/MS data was acquired for individual compounds as well as a mixture of compounds at a concentration of 1 µM in water using with the same acquisition methods as the study. All individual MS spectra in the figure are filtered for mass range event at 112.0505 m/z (cytosine). The identity of the source fragments was also confirmed by the corresponding MS/MS spectra. All standards are overlayed in a global range to allow for relative comparison. Cytosine (black), cytidine (red) and deoxycytidine (green) showed the strongest signal indicating good ionization in ESI+. CMP (dark blue) and dCMP (cyan) are also captured relatively well. CDP (yellow), CTP (purple), dCDP (gray) and dCTP (magenta) are not noticeable in this overlay because their intensity was extremely low. In general ribonucleotides and their deoxy relatives were better resolved in ESI-.


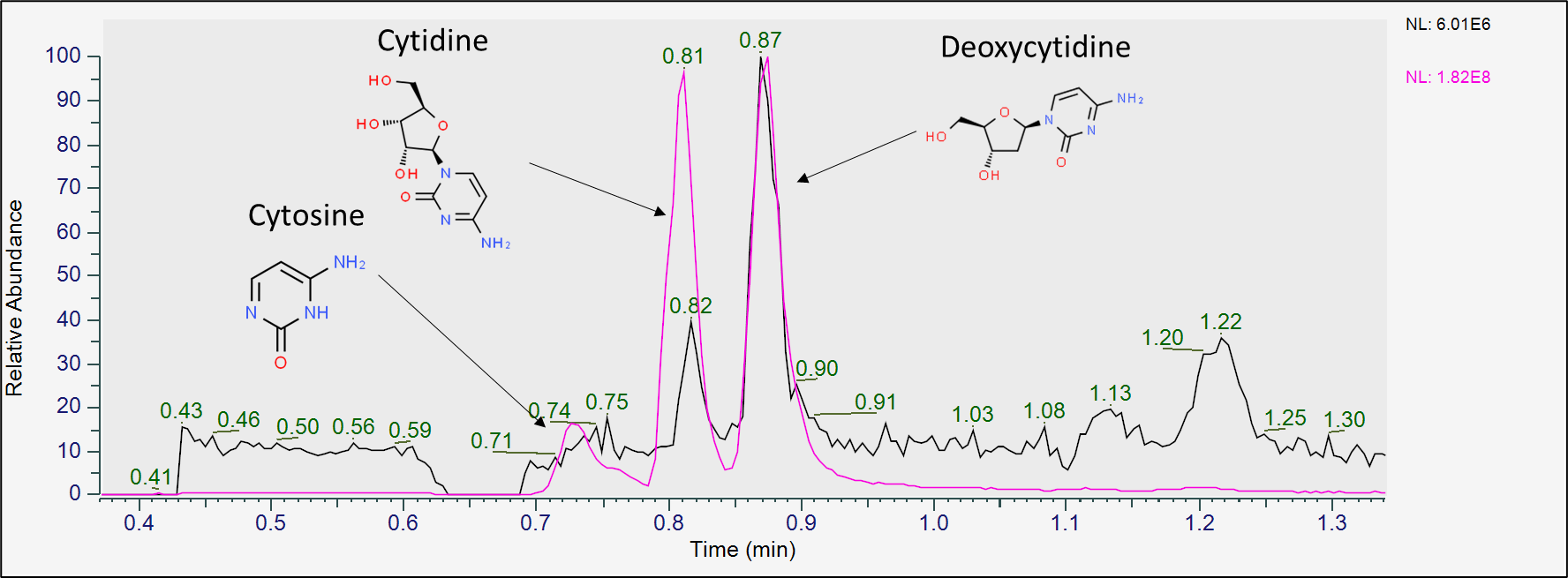


**Figure S10: Cytosine events in study serum with and without standards spiked in.** Study pooled serum (black) and study pooled serum with a 5 µM spike of cytosine-based nucleoside, ribonucleotides and deoxyribonucleotides standards (magenta) are overlayed in a local scale. The three peak cytosine patterns clearly indicate cytosine at ~0.7min, cytidine at ~0.81 min and deoxycytidine at ~0.87 min. It is important to note that the serum signa intensityl is at 6.01e6 and the spiked serum signal at 1.82e8.


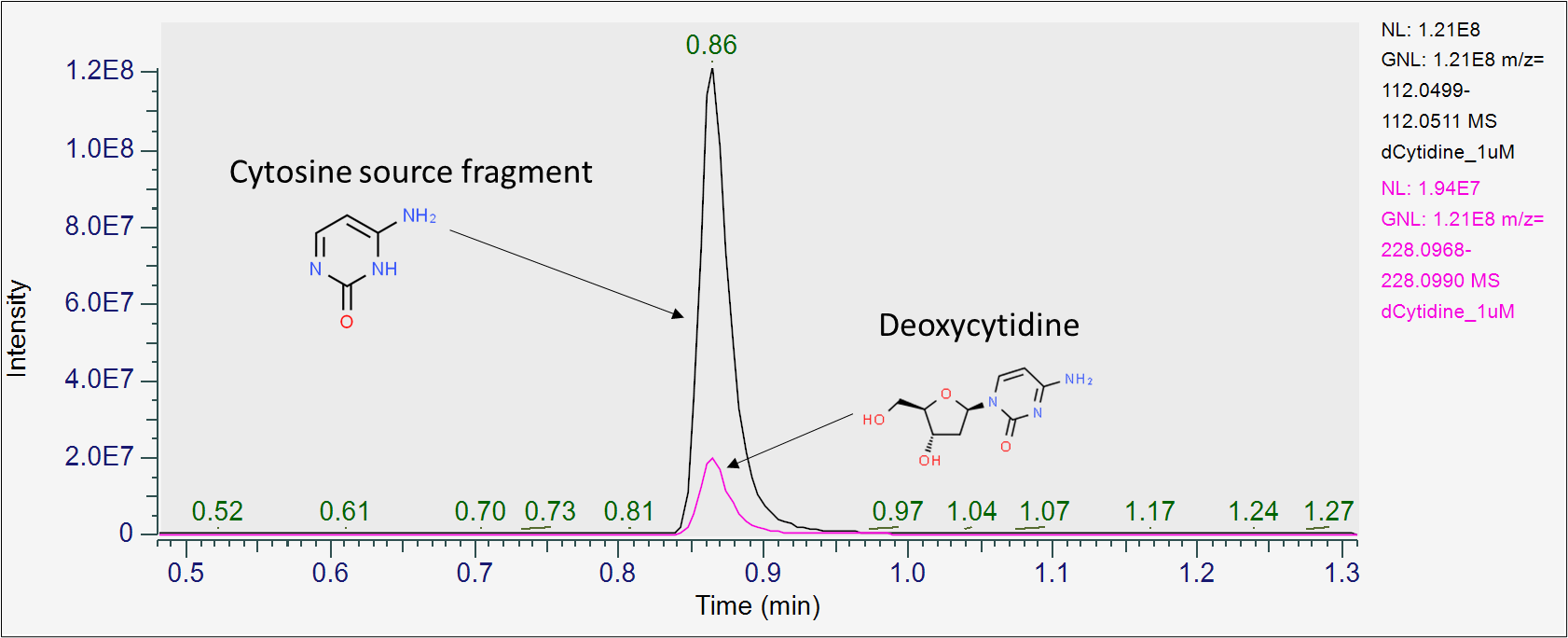


**Figure S11: Deoxycytidine ESI source fragmentation.** Shown are the extracted ion chromatogram of a 1µM solution of deoxycytidine in water filtered for cytosine event (black) and deoxycytidine event (magenta) at a global scale. The results shown demonstrate the deoxycytidine readily fragments at the source on the N-glycosidic bond between the cytosine and ribose moiety. Therefore, in the study we preferred to measure the cytosine event produced from the deoxycytidine. The overlap between both events in RT shows that the fragmentation is taking place at the ESI source.

# Supplementary discussion figures


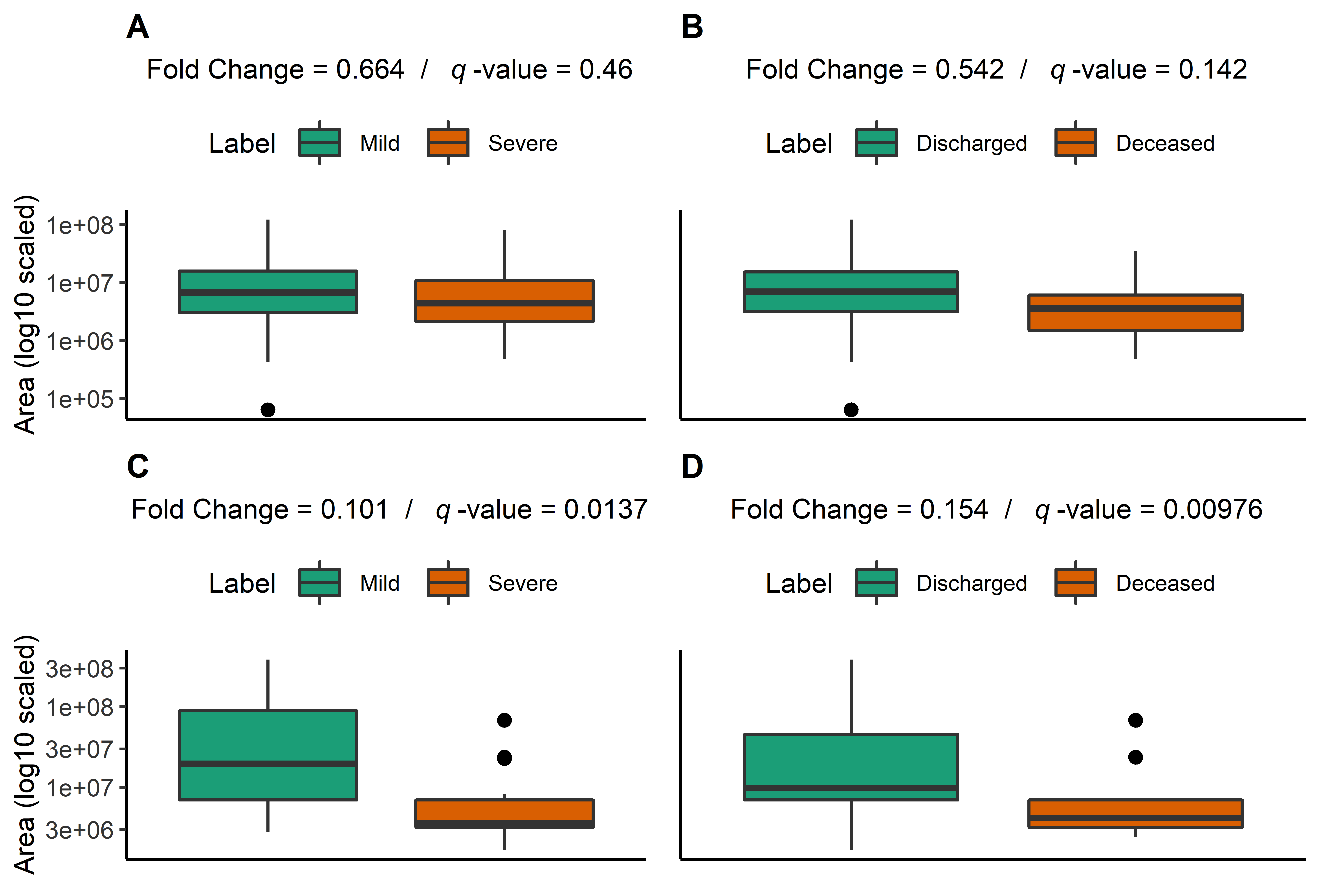


**Figure S12: Serum ergothioneine in patients based on (A) severity and (B) outcome, and serum piperine in severity (C) and outcome (D).** Ergothioneine shows expected trend (reduced in severe); however, falls short of q-value significance. The observed trend is stronger in poor outcome i.e. decreased levels of ergothioneine are more likely in poor outcome. Piperine levels appear significantly decreased in severe cases and poor outcome.

**Table S4: Injection order template.** Injection column contains the name and type of injection, followed by brief description of the nature, and use of the sample. ISTD column notes the samples that had addition of internal standards and finally analysed refers to the fact is the sample was analysed in the CD workflow or used for cleaning and conditioning of the system.

| **Injection** | **Description** | **ISTD** | **Analysed** |
| --- | --- | --- | --- |
| B1 | Blank for background exclusion |  | Yes |
| CQC1 | Conditioning samples |  |  |
| CQC2 |  |  |  |
| CQC3 |  |  |  |
| B2 | Blank for background exclusion and assessing carryover |  | Yes |
| CQC4 | Conditioning samples.  Not analysed, only used to condition the column before the study. |  |  |
| CQC5 |  |  |  |
| CQC6 |  |  |  |
| CQC7 |  |  |  |
| CQC8 |  |  |  |
| CQC9 |  |  |  |
| CQC10 |  |  |  |
| CQC11 |  |  |  |
| CQC12 |  |  |  |
| CQC13 |  |  |  |
| QC1 | Pool of the analytical batch samples. Used for normalization and metabolic feature quality check. |  | Yes |
| QC2 |  |  | Yes |
| QC3 |  |  | Yes |
| IQA1 | Inter-batch quality assurance. Used for inter-batch normalization. |  | Yes |
| Sample1 | Patient samples. Randomized.  Four samples per batch were analysed in duplicates.  30 patient samples were injected per analytical batch. |  | Yes |
| Sample2 |  |  | Yes |
| Sample3 |  |  | Yes |
| Sample4 |  |  | Yes |
| SQA1 | Commercial serum quality assurance, spiked with ISTD | Yes | Yes |
| QC4 | Pool of the batch samples |  | Yes |
| Sample5 | Patient samples. |  | Yes |
| Sample6 |  |  | Yes |
| Sample7 |  |  | Yes |
| Sample8 |  |  | Yes |
| Sample9 |  |  | Yes |
| IQA2 | Inter-batch quality assurance. Used for inter-batch normalization. |  | Yes |
| QC5 | Pool of the batch samples |  | Yes |
| Sample10 | Patient samples. |  | Yes |
| Sample11 |  |  | Yes |
| Sample12 |  |  | Yes |
| Sample13 |  |  | Yes |
| Sample14 |  |  | Yes |
| SQA2 | Commercial serum quality assurance, spiked with ISTD | Yes | Yes |
| QC6 | Pool of the batch samples |  | Yes |
| Sample15 | Patient samples. |  | Yes |
| Sample16 |  |  | Yes |
| Sample17 |  |  | Yes |
| Sample18 |  |  | Yes |
| Sample19 |  |  | Yes |
| IQA3 | Inter-batch quality assurance. Used for inter-batch normalization. |  | Yes |
| QC7 | Pool of the batch samples |  | Yes |
| Sample20 | Patient samples. |  | Yes |
| Sample21 |  |  | Yes |
| Sample22 |  |  | Yes |
| Sample23 |  |  | Yes |
| Sample24 |  |  | Yes |
| SQA3 | Commercial serum quality assurance, spiked with ISTD | Yes | Yes |
| QC8 | Pool of the batch samples |  | Yes |
| Sample25 | Patient samples. |  | Yes |
| Sample26 |  |  | Yes |
| Sample27 |  |  | Yes |
| Sample28 |  |  | Yes |
| Sample29 |  |  | Yes |
| IQA4 | Inter-batch quality assurance. Used for inter-batch normalization. |  | Yes |
| QC9 | Pool of the batch samples |  | Yes |
| Sample30 | Patient samples. |  | Yes |
| Sample31 |  |  | Yes |
| Sample32 |  |  | Yes |
| Sample33 |  |  | Yes |
| Sample34 |  |  | Yes |
| SQA4 | Commercial serum quality assurance, spiked with ISTD | Yes | Yes |
| QC10 | Pool of the batch samples |  | Yes |
| QC_ddMS1 | Pool of the batch samples for data dependent MS/MS in varying ranges and in triplicate (66-1000 m/z, 66-300 m/z, 300 - 600 m/z, 600 - 1000 m/z). |  | Yes |
| QC_ddMS2 |  |  | Yes |
| QC_ddMS3 |  |  | Yes |
| QC_ddMS4 |  |  | Yes |
| QC_ddMS5 |  |  | Yes |
| QC_ddMS6 |  |  | Yes |
| QC11 | Pool of the batch samples |  | Yes |
| QC_ddMS7 | Pool of the run samples for MS/MS in varying ranges and duplicates. |  | Yes |
| QC_ddMS8 |  |  | Yes |
| QC_ddMS9 |  |  | Yes |
| QC_ddMS10 |  |  | Yes |
| QC_ddMS11 |  |  | Yes |
| QC_ddMS12 |  |  | Yes |
| QC12 | Pool of the batch samples |  | Yes |
| IQA5 | Inter-batch quality assurance. Used for inter-batch normalization. |  | Yes |
| SQA5 | Commercial serum quality assurance, spiked with ISTD | Yes | Yes |
| QC13 | Pool of the run samples |  | Yes |
| QC14 |  |  | Yes |
| SQA50 | Commercial serum quality assurance, spiked with ISTD. Injected in varying volumes at 2.5, 3.75, 6.25, 7.5 µL in ESI+ and 5, 7.5, 12.5, 15 µL in ESI- | Yes | Yes |
| SQA75 |  | Yes | Yes |
| SQA125 |  | Yes | Yes |
| SQA150 |  | Yes | Yes |
| QC15 | Pool of the batch samples |  | Yes |
| QC16 |  |  | Yes |
| B3 | Blank for background exclusion and assess carryover |  | Yes |

# Supplementary methods and figures


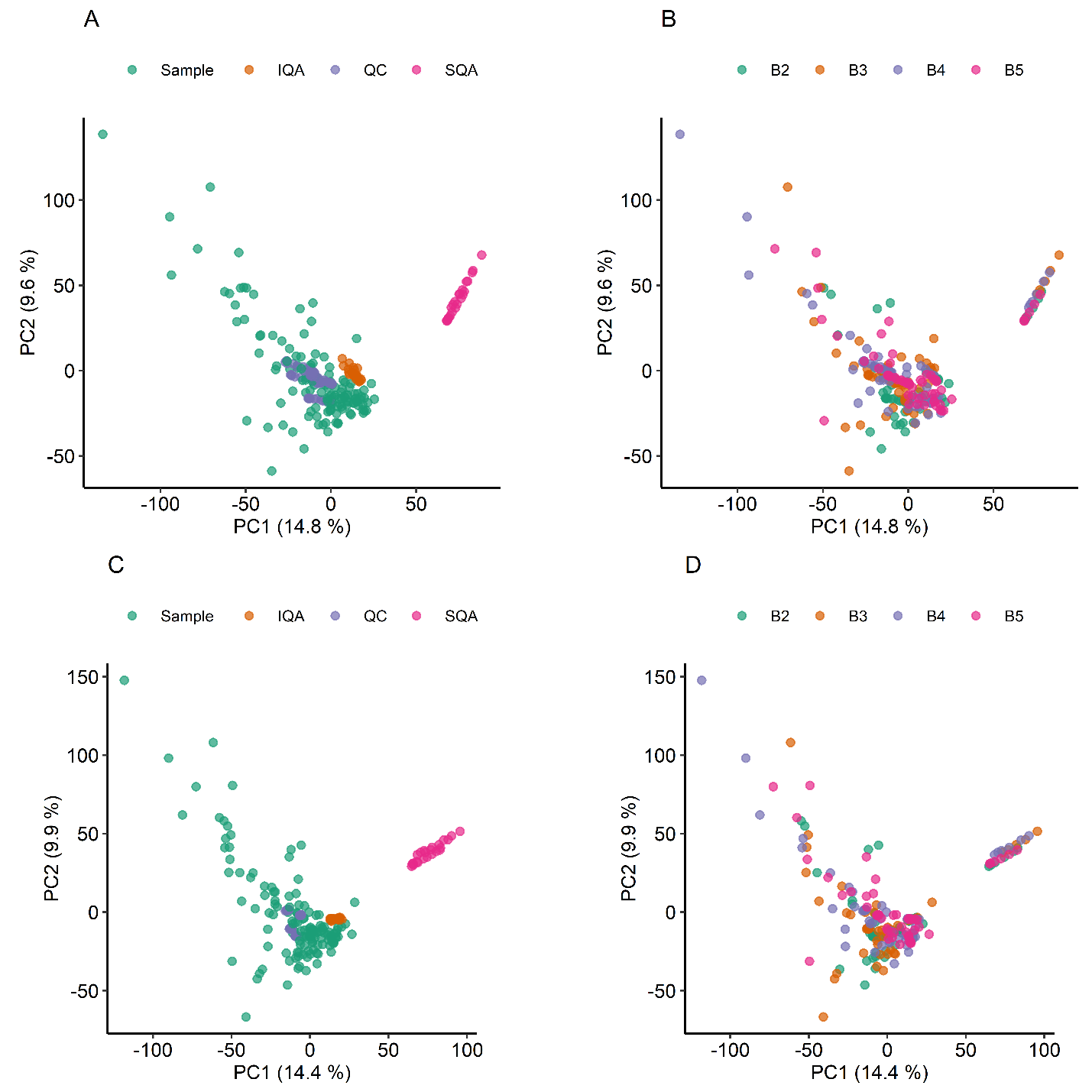


**Figure S13: PCA scores plot showing normalization results.** A and B show raw data coloured by group and batch, respectively. C and D plots show normalized data. QC samples in plot C do not overlap between batches as expected because they represent a pool of the samples in each batch therefore are different between batches. SQA samples show reduced but still present drift after normalization. This is due to the different nature of those samples as not all metabolic features present in the commercial serum were measured in the study samples (e.g. recreational drugs). Finally, batches after normalization show a healthy distribution with no visible batch effect. Axes are labelled with principal component (PC) and its explained variance in percentage.

## MS acquisition settings for the analysis of longitudinal serum samples using an ID-X Tribrid mass spectrometer

As described in the main text, serum samples for longitudinal untargeted metabolomics were acquired in the same manner as baseline samples except a ThermoFisher Scientific ID-X Tribrid mass spectrometer was used. Instrument parameters are provided below.

Full-scan MS data was acquired in the Orbitrap mass analyser in the m/z range 66.7-1,000 with a mass resolution of 120,000 Full Width Half Maximum (FWHM) at m/z = 200, a chromatographic peak width of 4 s (FWHM), Normalized AGC target (%) = 50 and maximum injection time = 100 ms. Internal mass calibration was used in negative ionization mode only. Source and ion transfer parameters applied were as follows: Sheath flow rate (arbitrary units) = 40, auxiliary gas flow rate (arbitrary units) = 8, sweep gas flow rate (arbitrary units) = 1, spray voltage = 3.5 kV (positive) and -3.0 kV (negative), capillary temperature (°C) = 275, S-lens RF level (%) = 45, auxiliary gas heater temperature (°C) 300, source position = M 2.

Data-dependent MS/MS (ddMS^2^) data acquisition was performed on pooled samples at the end of the analytical batch as described in guidelines by (Broadhurst et al., 2018). In a manner similar to that proposed by (Mullard et al., 2015), ddMS^2^ data acquisition was performed on 4 precursor mass ranges in triplicate injections: 66.7-1,000, 66.7-300, 300-600 and 600-900. Data was acquired in the Orbitrap mass analyser with a mass resolution of 60,000 Full Width Half Maximum (FWHM) at m/z = 200 for the master scan and 30,000 FWHM at m/z 200 for ddMS^2^ , both with a chromatographic peak width to 4 s (FWHM), Normalized AGC target (%) = 50, maximum injection time = 54 ms, cycle time (sec) = 0.6 , isolation window = 1.5 m/z, stepped HCD = 20, 40 and 60, AGC target Standard, intensity threshold = 2x10^4^, exclude isotopes = on and dynamic exclusion = 6.0 s.

The ThermoFisher Scientific AcquireX deep scan acquisition workflow was also employed here on pooled samples for each batch of longitudinal serum samples. This workflow enables an exhaustive acquisition of unique features in samples by using automated and iterative data dependent MS/MS acquisition, using automatically updated run-to-run inclusion and exclusion lists.

# References

Ansone, L., Ustinova, M., Terentjeva, A., Perkons, I., Birzniece, L., Rovite, V., Rozentale, B., Viksna, L., Kolesova, O., Klavins, K.*, et al.* (2021). Tryptophan and arginine metabolism is significantly altered at the time of admission in hospital for severe COVID-19 patients: findings from longitudinal targeted metabolomics analysis. medRxiv, 2021.2003.2031.21254699.

Blasco, H., Bessy, C., Plantier, L., Lefevre, A., Piver, E., Bernard, L., Marlet, J., Stefic, K., Benz-de Bretagne, I., Cannet, P.*, et al.* (2020). The specific metabolome profiling of patients infected by SARS-COV-2 supports the key role of tryptophan-nicotinamide pathway and cytosine metabolism. Scientific Reports *10*, 16824.

Broadhurst, D., Goodacre, R., Reinke, S.N., Kuligowski, J., Wilson, I.D., Lewis, M.R., and Dunn, W.B. (2018). Guidelines and considerations for the use of system suitability and quality control samples in mass spectrometry assays applied in untargeted clinical metabolomic studies. Metabolomics *14*, 72.

Doğan, H.O., Şenol, O., Bolat, S., Yıldız, Ş.N., Büyüktuna, S.A., Sarıismailoğlu, R., Doğan, K., Hasbek, M., and Hekim, S.N. (2021). Understanding the pathophysiological changes via untargeted metabolomics in COVID‐19 patients. Journal of medical virology *93*, 2340-2349.

Kimhofer, T., Lodge, S., Whiley, L., Gray, N., Loo, R.L., Lawler, N.G., Nitschke, P., Bong, S.-H., Morrison, D.L., Begum, S.*, et al.* (2020). Integrative Modelling of Quantitative Plasma Lipoprotein, Metabolic and Amino Acid Data Reveals a Multi-organ Pathological Signature of SARS-CoV-2 Infection. Journal of Proteome Research.

López-Hernández, Y., Monárrez-Espino, J., Herrera-van Oostdam, A.-S., Delgado, J.E.C., Zhang, L., Zheng, J., Valdéz, J.O., Mandal, R., González, F.O., and Borrego, J.C. (2021). Targeted Metabolomics Identifies High Performing Diagnostic and Prognostic Biomarkers for COVID-19.

Mullard, G., Allwood, J.W., Weber, R., Brown, M., Begley, P., Hollywood, K.A., Jones, M., Unwin, R.D., Bishop, P.N., Cooper, G.J.S.*, et al.* (2015). A new strategy for MS/MS data acquisition applying multiple data dependent experiments on Orbitrap mass spectrometers in non-targeted metabolomic applications. Metabolomics *11*, 1068-1080.

Overmyer, K.A., Shishkova, E., Miller, I.J., Balnis, J., Bernstein, M.N., Peters-Clarke, T.M., Meyer, J.G., Quan, Q., Muehlbauer, L.K., and Trujillo, E.A. (2020). Large-Scale Multi-omic Analysis of COVID-19 Severity. Cell systems.

Páez-Franco, J.C., Torres-Ruiz, J., Sosa-Hernández, V.A., Cervantes-Díaz, R., Romero-Ramírez, S., Pérez-Fragoso, A., Meza-Sánchez, D.E., Germán-Acacio, J.M., Maravillas-Montero, J.L., Mejía-Domínguez, N.R.*, et al.* (2021). Metabolomics analysis reveals a modified amino acid metabolism that correlates with altered oxygen homeostasis in COVID-19 patients. Scientific Reports *11*, 6350.

Shen, B., Yi, X., Sun, Y., Bi, X., Du, J., Zhang, C., Quan, S., Zhang, F., Sun, R., Qian, L.*, et al.* (2020). Proteomic and Metabolomic Characterization of COVID-19 Patient Sera. Cell *182*, 59-72.e15.

Sindelar, M., Stancliffe, E., Schwaiger-Haber, M., Anbukumar, D.S., Albrecht, R.A., Liu, W.-C., Travis, K.A., García-Sastre, A., Shriver, L.P., and Patti, G.J. (2021). Longitudinal Metabolomics of Human Plasma Reveals Robust Prognostic Markers of COVID-19 Disease Severity. medRxiv, 2021.2002.2005.21251173.

Sumner, L.W., Amberg, A., Barrett, D., Beale, M.H., Beger, R., Daykin, C.A., Fan, T.W.M., Fiehn, O., Goodacre, R., Griffin, J.L.*, et al.* (2007). Proposed minimum reporting standards for chemical analysis. Metabolomics *3*, 211-221.

Thomas, T., Stefanoni, D., Reisz, J.A., Nemkov, T., Bertolone, L., Francis, R.O., Hudson, K.E., Zimring, J.C., Hansen, K.C., Hod, E.A.*, et al.* (2020). COVID-19 infection alters kynurenine and fatty acid metabolism, correlating with IL-6 levels and renal status. JCI Insight *5*.

Wu, D., Shu, T., Yang, X., Song, J.-X., Zhang, M., Yao, C., Liu, W., Huang, M., Yu, Y., Yang, Q.*, et al.* (2020). Plasma metabolomic and lipidomic alterations associated with COVID-19. National Science Review *7*, 1157-1168.
